# Analysis of the core bacterial community associated with consumer-ready Eastern oysters (*Crassostrea virginica)*

**DOI:** 10.1371/journal.pone.0281747

**Published:** 2023-02-22

**Authors:** Ian S. Hines, Justin Markov Madanick, Stephen A. Smith, David D. Kuhn, Ann M. Stevens

**Affiliations:** 1 Department of Biological Sciences, Virginia Tech, Blacksburg, Virginia, United States of America; 2 Center for Emerging, Zoonotic, and Arthropod-borne Pathogens, Virginia Tech, Blacksburg, Virginia, United States of America; 3 Department of Biomedical Sciences and Pathobiology, Virginia Tech, Blacksburg, Virginia, United States of America; 4 Department of Food Science and Technology, Virginia Tech, Blacksburg, Virginia, United States of America; Bigelow Laboratory for Ocean Sciences, UNITED STATES

## Abstract

Shellfish, such as the Eastern oyster (*Crassostrea virginica*), are an important agricultural commodity. Previous research has demonstrated the importance of the native microbiome of oysters against exogenous challenges by non-native pathogens. However, the taxonomic makeup of the oyster microbiome and the impact of environmental factors on it are understudied. Research was conducted quarterly over a calendar year (February 2020 through February 2021) to analyze the taxonomic diversity of bacteria present within the microbiome of consumer-ready-to-eat live Eastern oysters. It was hypothesized that a core group of bacterial species would be present in the microbiome regardless of external factors such as the water temperature at the time of harvest or post-harvesting processing. At each time point, 18 Chesapeake Bay (eastern United States) watershed aquacultured oysters were acquired from a local grocery store, genomic DNA was extracted from the homogenized whole oyster tissues, and the bacterial 16S rRNA gene hypervariable V4 region was PCR-amplified using barcoded primers prior to sequencing via Illumina MiSeq and bioinformatic analysis of the data. A core group of bacteria were identified to be consistently associated with the Eastern oyster, including members of the phyla Firmicutes and Spirochaetota, represented by the families *Mycoplasmataceae* and *Spirochaetaceae*, respectively. The phyla Cyanobacterota and Campliobacterota became more predominant in relation to warmer or colder water column temperature, respectively, at the time of oyster harvest.

## Introduction

The Eastern oyster, *Crassostrea virginica*, has historically been an important agricultural commodity in the Chesapeake Bay area (eastern United States). Heightened demand, decreased wild-caught supply and increased profit margins have contributed to an expanding interest in aquaculture-based methodologies to cultivate oysters [1]. Aquaculture processes may include genetic breeding, maintenance and/or collection of animals in a controlled fashion. Overall, the aquaculture industry for a variety of aquatic organisms has grown faster than any other sector in food production globally since the 1980s [2]. The aquaculture industry as a whole is projected to grow from $1 billion U.S. dollars (USD) to $3 billion USD by 2025, the value of the United States shellfish industry should follow a similar trajectory [3].

In 2011, the National Shellfish initiative was created by the National Oceanic and Atmospheric Administration (NOAA) to emphasize the need for updated and streamlined aquaculture policies [4, 5]. Consequently, several states launched shellfish initiatives which aimed to allocate resources and funding towards special planning, permitting, and research [6, 7]. In the Chesapeake Bay area over 6,000 acres of brackish water was expediently permitted in Virginia and Maryland to cultivate oysters [4]. Better understanding the role of the environmental and host-associated microbiome is important to support expansion of the oyster aquaculture industry. The water column in which oysters reside is a microorganism-rich environment which inevitably impacts both the oyster population, as well as the food safety of the output product, as high-value oysters are consumed live. Thus, the microbiome present in consumer-ready oysters has a direct impact on human health. Modifying the microbial composition of oysters through the application of either antibiotics or probiotics (i.e., live beneficial microorganism) can help farmers prevent disease outbreak in oysters during aquaculture production [8] and in humans consuming raw oysters. However, antibiotic treatments have been banned in the United States and are diminishing in use by the aquaculture industry as a whole. Therefore, investigations into the efficacy of probiotics and prebiotic treatments (i.e., compounds used to support beneficial microbial metabolism and growth) have become more common.

The probiotic organisms *Phaeobacter* sp. S4 or *Bacillus pumilus* RI06-95 have been shown to decrease oyster death when probiotic-treated animals were challenged with the oyster pathogens *Vibrio tubiashii* or *Roseovarius crassostreae*, indicating the role of a probiotic-supplemented microbiome in host-pathogen defense [9, 10]. Likewise, key microbiome constituents of the Pacific oyster (*Crassostrea gigas*), *Winogradskyella* spp. and *Bradyrhizobiaceae* spp. confer high resistance to disease caused by the OsHV-1 μvar virus, which is responsible for high mortality rates in oysters [11]. With regard to human health, oysters are the main vector of *Vibrio parahaemolyticus* infections [12], the number one source of seafood-borne gastroenteritis in the world. Due to the inherent significance of the oyster microbiome in protecting the animals from pathogen challenge [13], it is important to better define the endogenous microbiome of *Crassostrea virginica* to establish a rationale for improvements to probiotic supplementation as a disease prevention strategy.

Characterization of aquaculture-raised animal microbiomes over longitudinal studies can also provide critical information describing the conditions most suitable for bacterial growth, including human pathogens [14–16]. Some literature has indicated the ambient water column temperature is a key factor driving the fluctuations of the core microbiome of Eastern oysters [17]. For example, temperature-dependent bacterial carbon source-substrate utilization may lead to fluctuations in the microbial populations [18]. Elevated temperatures, in addition to elevated carbon dioxide concentrations, have also been shown to increase microbial diversity and richness in oysters [15]. Temperature-driven stress has even been shown to alter surviving oyster host microbiome to a greater extent than infection by pathogens [19].

Therefore, this study sought to identify the bacterial communities within the microbiome of consumer-ready Eastern oysters that persist in the processed animals independent of seasonal water temperature. Firmicutes and Spirochaetota were identified as core bacteria phyla present for the duration of the year. Cyanobacterota and Campliobacterota were more dominant when harvested from warmer (summer) or colder (winter) water, respectively, in comparison to more moderate temperatures. Diversity analyses indicated greater bacterial community diversity with warmer water temperature, and more similar composition with colder water temperature. The results presented here have defined some resilient core bacterial components of the microbiome of Eastern oysters and may help guide strategies for improved oyster host-microbe interactions.

## Methods

### Oyster samples

Eighteen consumer-ready market size oysters per sampling event, were originally harvested from one region of the north-central Chesapeake Bay, including tributary rivers (S1 Table). The oysters were purchased from one wholesaler either directly or through a local grocery store chain on five different dates spread out roughly quarterly over a calendar year (February, June, August and November 2020 to February 2021; 90 animals total). A desired May 2020 sampling was delayed until June due to COVID-19 pandemic-related supply chain issues. Purchased animals were from two brands that are marketed by the same wholesaler company, which performed the post-harvesting processing procedures. Oysters were all similarly processed by washing, rinsing the outside of the animals and then storing them in refrigeration or ice until they were purchased. Oysters remained closed during processing due to external stresses. Specific oyster source locations were obtained from the company shipment labels. Using these locations, environmental conditions, including temperature, salinity and dissolved oxygen, during the time of oyster collection were recorded (S1 Table) using the NOAA buoy database [20] for the two buoys that were closest to the harvest sites. It was not possible to obtain water column samples for analysis.

### Genomic DNA extraction

For each timepoint, 18 oysters were shucked and an OMNI homogenizer (Omni International, Kennesaw, GA) was used to homogenize each whole oyster using ethanol-sanitized equipment. Genomic DNA was isolated from tissue homogenates using the Qiagen PowerSoil kit (Qiagen, Germantown, MD) per the manufacturer’s protocol with alterations including a 10-min incubation at 72°C after addition of C1 buffer and a 5-min incubation at 72°C prior to elution in 50 μL dH_2_O. Tissue homogenates were added to the PowerBead (Qiagen) tubes using a range of weights between 10 and 60 mg. Prior to storage at -20°C, the quantity and purity (i.e., A_260_ /A_280_ and A_260_ /A_280_) of the gDNA samples were analyzed via a nanospectrophotometer (Implen, Westlake Village, CA).

### 16S rRNA gene PCR amplification and purification

The purified gDNA obtained from oyster homogenized tissues was used as the template for PCR-amplification of the bacterial 16S rRNA gene V4 region. PCR conditions for each reaction were as follows: 24.5 μL of 1X Q5 Master Mix (New England Labs, Ipswich, MA) containing 500 nM each of forward universal barcoded primer (515f; AATGATACGGCGACCACCGAGATCTACACGCTxxxxxxxxxxxxTATGGTAATTGTGTGYCAGCMGCCGCGGTAA, with the x region representing the golay barcode) and reverse primer (806r; CAAGCAGAAGACGGCATACGAGATAGTCAGCCAGCCGGACTACNVGGGTWTCTAAT), 30 ng of gDNA template and a variable amount of dH_2_O to bring the total volume up to 25 μL. PCR amplification of each sample was performed in triplicate along with one negative water control per reaction. The universal barcoded forward primers were created according to Caporaso et al. [21]. Thermal cycler (Bio-Rad, Hercules, CA) program settings were as follows: initial denaturation at 98°C for 30 sec; 30 cycles of denaturation at 98°C for 10 sec, annealing at 50°C for 20 sec, and elongation at 72°C for 15 sec; final elongation at 72°C for 2 min.

The triplicate PCR-amplified samples were pooled together and visualized on a 1% agarose gel to confirm product size. Each pooled triplicate PCR product was subsequently purified using the Qiagen PCR purification kit with the following alterations: 50 μl of dH_2_O was used for the final elution and a 5-minute incubation at 72°C prior to final elution. Following PCR purification, amplicons were analyzed for yield and purity using Qubit fluorometry.

### Sequencing and bioinformatics

Purified V4 amplicons were then processed via Solid Phase Reversible Immobilization (SPRI) beads to remove unwanted lower molecular weight bands (Genomics Sequencing Center, Fralin Life Sciences Institute, Virginia Tech, Blacksburg, VA). SPRI bead-purified amplicons were further processed using the Pippin Prep (Sage Science, Beverly, MA) to filter out contaminating higher molecular weight bands associated with host 18S rDNA while purifying the V4 bands of interest. Amplicons were then sequenced on the Illumina MiSeq platform using 500 cycles of 250 bp paired-end sequencing at a concentration of 8.5 pM. Phix was spiked in at 25% to analyze run efficiency.

The sequences generated from MiSeq were analyzed for microbial diversity and identification using Quantitative Insights Into Microbial Ecology (QIIME2 v. 2020.2; [22]). Sequences were denoised using the DADA2 program [23] to generate amplicon sequence variants (ASVs). ASVs were further filtered to remove eukaryotic-associated sequences. Filtered ASVs were assigned taxonomy using a 16S V4 region-specific classifier based on the SILVA database, version 138 [24–26]. Taxonomically-assigned ASVs at each time point were pooled (n = 18 animals) and used to create taxonomic barplots by collapsing them to their shared Phylum and Family levels. Individual ASVs were also used for diversity analyses within the R statistics program [27] using the following packages: qiime2R v.0.99.6 [28], phyloseq v.1.27.6 [29], vegan v.2.5–7 [30], ggplot2 v.3.3.5 [31], complexheatmap v.2.9.1 [32]. Phylum-level plots used only the 100 most abundant ASVs for visualization which represented at least 95% of the total microbiome for each season.

The demultiplexed paired-end sequences associated with each of the 90 animals have been uploaded to the NCBI Sequence Read Archive (SRA) database under the BioProject ID PRJNA845766.

### Statistics

The overall bacterial community diversities for the oysters within each season (i.e., alpha diversities as analyzed by Shannon, evenness, and observed ASVs) were calculated via QIIME2. Differences between alpha diversities were analyzed for statistical significance using the non-parametric Kruskal-Wallis test with Dunn post-hoc test to identify pairwise significance. The similarities between the different bacterial community samples at each season (i.e., beta diversity) were analyzed using weighted and unweighted UniFrac metrics. Beta diversities were further analyzed for statistical significance using the permutational multivariate analysis of variance (PERMANOVA) followed by pairwise comparisons for the five samples within the vegan package of R (RStudio, Boston, MA). Significant shifts in individual taxa with respect to harvest season were identified via Analysis of Compositions of Microbiomes with Bias Correction (ANCOM-BC) [33].

## Results

### Taxonomic identification

**Fig 1:**
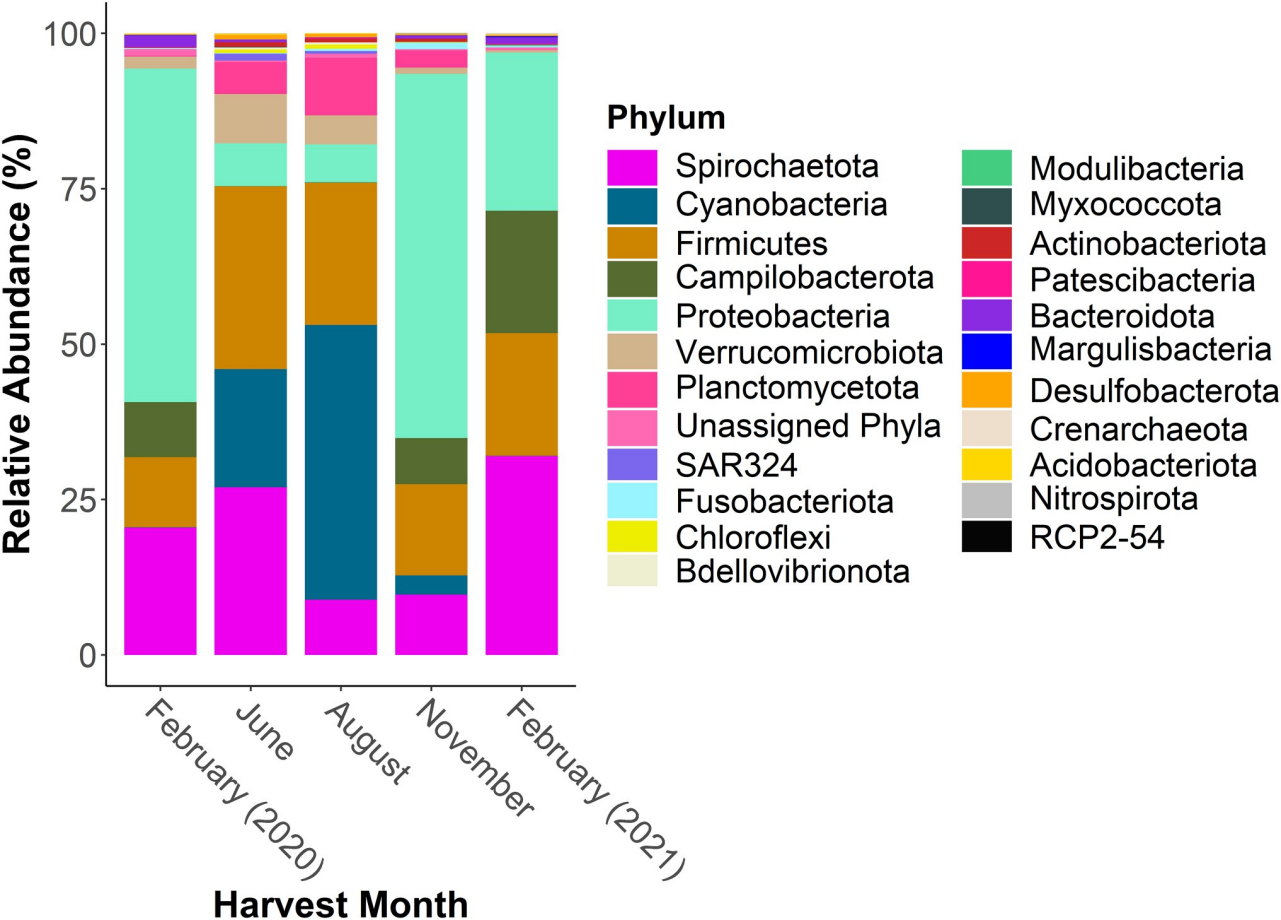
Phylum-level relative abundances within the Eastern oyster bacterial community across time. Eastern oysters were collected quarterly over a year (n = 18 oysters per season) including a second harvest one year following the first harvest. The 16S rRNA gene V4 region was sequenced from the oyster homogenates wherein the bacterial communities were examined. The average relative abundance of the top phyla comprising at least 95% of the total bacterial community for all treatment groups are represented in the plot.

**Fig 2:**
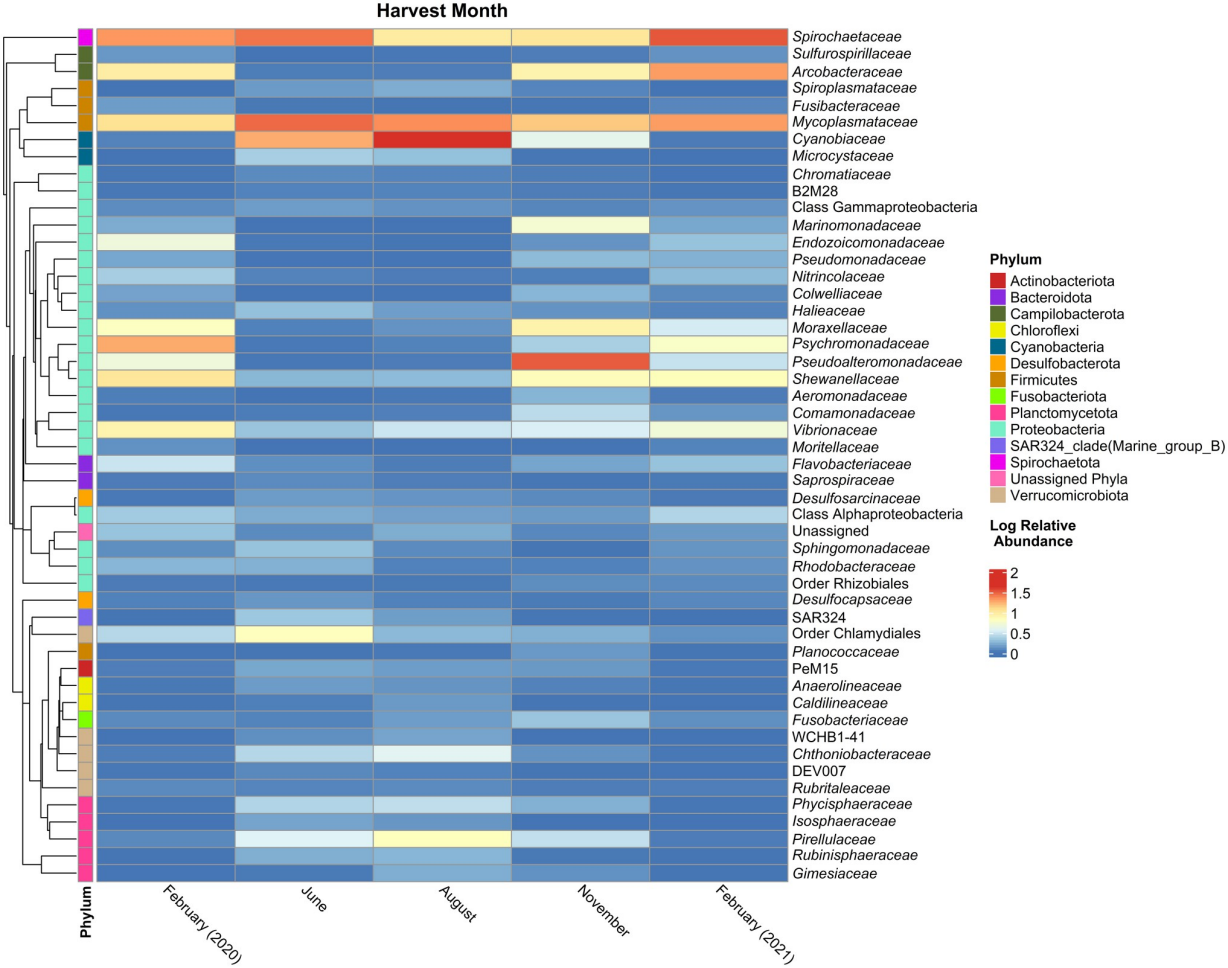
Family-level relative abundances within the Eastern oyster bacterial community across time. Oyster-isolated 16S rRNA V4 region amplicons were sequenced via Illumina MiSeq. The average log relative abundance of the top 50 family-level taxa are represented in the plot for each harvest date. The phylogenetic tree was constructed via QIIME2 to denote the phylogeny and phylum to which each family-level taxon belongs.

A total of 9,883,610 sequences were generated from MiSeq for bioinformatics analysis. After filtering the data 7,050,516 sequences were characterized as 1,659 unique ASVs. Several shifts in the bacterial communities were observed with respect to the time of harvest. For example, bacteria associated with the phyla Campilobacterota and Proteobacteria appear to have higher relative abundances during November and February (colder water column temperatures) and greatly reduced levels during the June and August (warmer water column temperatures) (Fig 1) with individual animals showing similar trends (S1 Fig). The most dominant family of Campilobacterota, *Arcobacteraceae* (Fig 2, S2 Fig), is represented by *Pseudarcobacter* spp. This organism is absent from the top 50 taxa of the June and August animals, but it is present at ~6% and 15% relative abundance within the February 2020 and 2021 animals, respectively (S2 Table). Conversely, Cyanobacteria, represented by *Cyanobium* sp. within the *Cyanobiaceae* family, are at the highest relative abundance during June and August and virtually absent during February (Figs 1 and 2, S2 Table, S2 Fig).

Apart from the shifts in bacterial constituents correlated with temporal collection times, several taxa remain present throughout the year. For example, the phyla Firmicutes and Spirochaetota (Fig 1, S1 Fig), specifically the families *Mycoplasmataceae* and *Spirochaetaceae* (Fig 2, S2 Fig), appear to consistently be components of the oyster bacterial community across all seasons. *Mycoplasma* spp. (within the *Mycoplasmataceae* family) specifically constitute no less than 10% of the total oyster bacterial community at all samplings (S2 Table). Similar to *Mycoplasma* sp., an unidentified organism within the *Spirochaetaceae* family, on average constitutes ~18% of the total bacterial community throughout the year (S2 Table). Other organisms consistently present, with an average relative abundance of less than 3% (S2 Table), included an unidentified organism associated with the Order Chlamydiales (~2%), an unidentified organism associated with the Class Alphaproteobacteria (~1%), *Halioglobus* spp. (~0.3%), and an unidentified organism associated with the Class Gammaproteobacteria (~0.3%).

### Diversity analyses

Alpha diversities of the oyster-associated bacterial communities were calculated via the Shannon metric (H), the evenness metric, and observed ASVs (Table 1). Regardless of the metric used, the overall diversities associated with oysters were highest in August. The lowest H diversities associated with oysters were identified in the November and February sampling periods, which are all highly similar. Though statistically similar to February 2020 oysters, the H diversities of June oysters were significantly higher than the February 2021 oysters (P < 0.05). The highest number of observed ASVs was in the August oysters (~219 ASVs) which was similar to June oysters (~202 ASVs). The number of observed ASVs measured during August and June was significantly higher than in oysters collected during the November and February (P < 0.05). Higher values resulting from the evenness metric calculations showed that August and February 2020 oysters were well-represented bacterial communities (i.e., not dominated by just a few taxa). February 2021 oysters exhibited the lowest evenness over the course of a year, moreover, the evenness was significantly lower than oysters collected during August (P < 0.001). In comparison, oysters collected during November and June exhibited highly similar evenness measurements. While not statistically different, the year-over-year alpha diversities calculated from the February 2020 and February 2021 oysters, respectively, indicated a decrease in each diversity metric.

**Table 1:** Temporal correlation to oyster microbiome alpha diversities.

Season	Shannon (H)	Evenness	Observed ASVs
February (2020)	4.24 ± 0.52^a^^b^	0.61 ± 0.06^a^^b^	137 ± 51^a^
June	4.36 ± 1.01^a^	0.57 ± 0.11^a^	202 ± 57^b^
August	5.30 ± 0.64^c^	0.68 ± 0.06^b^	219 ± 48^b^
November	3.92 ± 1.06^a^^b^	0.56 ± 0.12^a^	136 ± 57^a^
February (2021)	3.46 ± 0.56^b^	0.52 ± 0.07^a^	108 ± 29^a^

^abc^ Different superscript letters represent P < 0.05 between seasons within a given diversity metric following one-way Kruskal Wallis with Dunn post-hoc test.

**Fig 3:**
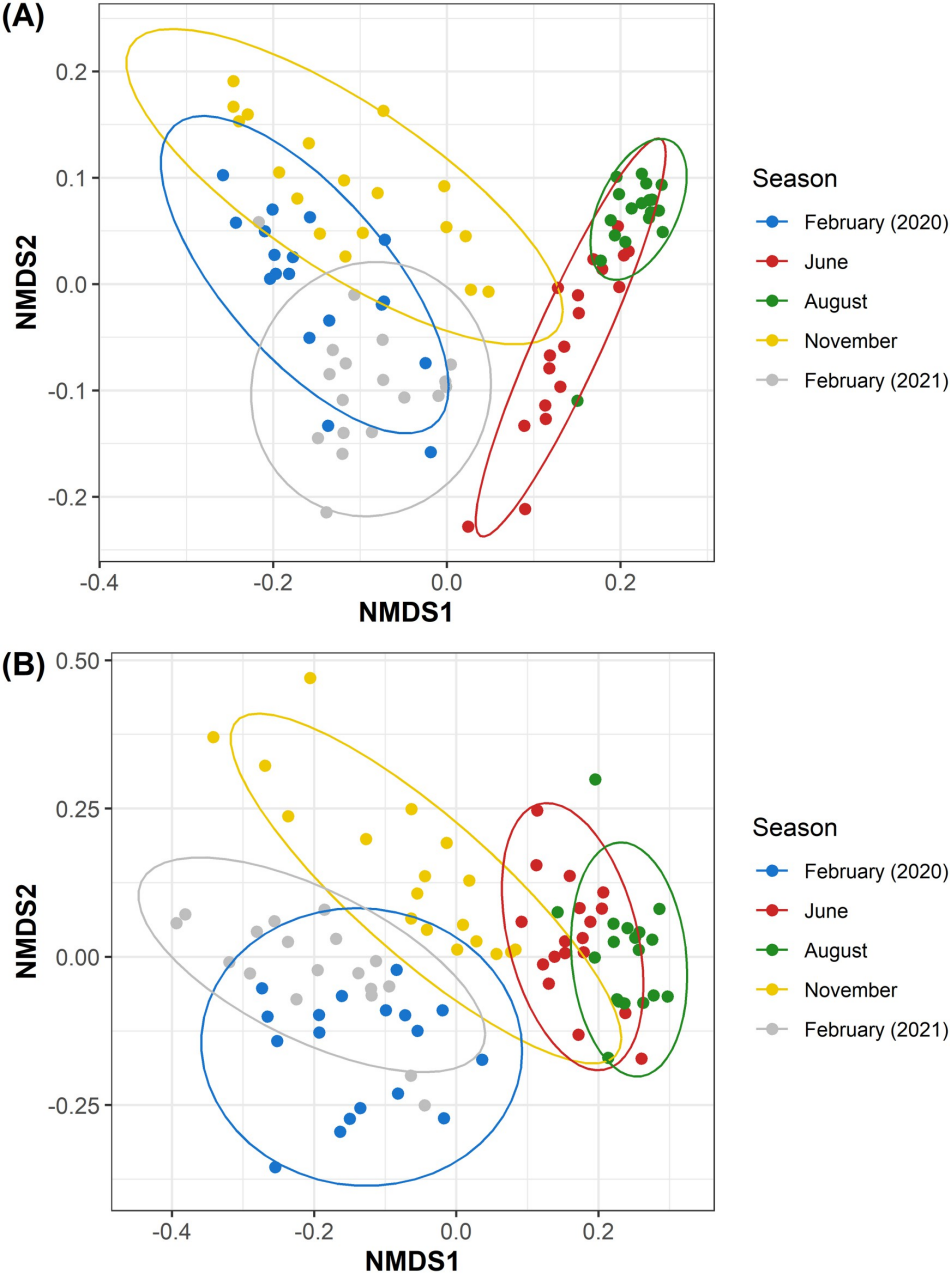
Eastern oyster bacterial community beta diversities correlated with time of harvest. Non-metric multidimensional scaling was used to visualize the dissimilarities between seasonal bacterial communities of the Eastern oyster. Dissimilarities were calculated via (A) unweighted UniFrac and (B) weighted UniFrac beta diversity metrics.

Bacterial communities between oysters harvested at each season were compared for similarities both without and with relative abundance taken into account using the unweighted UniFrac (Fig 3A) and weighted UniFrac metrics (Fig 3B), respectively. Though oysters harvested during June and August appeared to share the most taxa, a PERMANOVA indicated the two groups of bacterial communities were significantly different (p < 0.01). In fact, the PERMANOVA indicated the bacterial communities were significantly different between every season (Fig 3). Even the year-over-year analysis indicated statistically different bacterial communities from the oysters collected during the two February time points regardless of UniFrac metric.

Differentially abundant taxa identified via ANCOM-BC encompassed many bacterial families including those highlighted above: *Mycoplasmataceae*, *Spirochaetaceae*, *Cyanobiaceae*, and *Arcobacteraceae* (S2 Table). Although the taxa associated with families such as *Mycoplasmataceae* and *Spirochaetaceae* change significantly over the course of year, they are more consistently present (Fig 1, S2 Table) compared to taxa associated within the *Cyanobiaceae* and *Arcobacteraceae* families. The latter two groups of taxa exhibit more dramatic shifts in relative abundance over the course of the year (Fig 1, S2 Table).

## Discussion

In addition to providing several key ecological benefits to the Chesapeake Bay ecosystem, Eastern oysters are also an important economic resource to the coastal regions of the Chesapeake Bay in the United States [34, 35]. Studies about the microbiome of oysters are important to improving sustainable aquaculture practices, as the microbiome provides several key benefits for the host and is considered akin to a vital organ of many animals. The structure of the microbiome has been studied in numerous invertebrate hosts such as insects, corals [36, 37], crustaceans [38, 39], and other bivalves [40, 41]. These studies illustrate the functional necessity for a healthy, stable microbiome for invertebrates, including the Eastern oysters examined here.

A group of bacteria that is present in a population of host animals regardless of external factors may constitute components of the core microbiome. Many of the external factors impacting the bacterial community are environmental in nature, such as the temperature and salinity of the water column. However, some are anthropogenic in nature. It is a common practice to process oysters following harvest to make them more “attractive” to consumers by removing loose external material and to make them safer for human consumption by reducing the infection potential by bacteria such as *Vibrio* spp. [42, 43]. Importantly, the consumer ready-to-eat oysters used for this analysis were exposed to post-harvest methods (two brands marketed and processed after their harvest by the same wholesaler company). Therefore, the bacteria most resilient to the post-harvest treatments were the ones identified through the community profile analysis.

Within the oysters studied here, a core group of bacteria was found to reside in the microbiome at relatively consistent relative abundances throughout the year (i.e., Firmicutes and Spirochaetota; Fig 1 and S1 Fig). *Mycoplasmataceae* and *Spirochaetaceae* are the two most prevalent families present in oysters throughout the year in consumer-ready oysters (Fig 2 and S2 Fig). This suggests that these bacteria have a more permanent relationship with the oysters and that they are resilient to changing environmental conditions including water temperature fluctuations and stressful post-harvesting procedures. Interestingly, these findings align with a number of *in situ* studies as discussed below.

Within the *Mycoplasmataceae* family, *Mycoplasma* spp. were identified as the most common species (S2 Table) observed in the post-harvest oysters. *Mycoplasma* spp. lack cell walls and have often been found to associate with a host organism, including oysters based on *in situ* analysis [44–49]. Some of them also represent a pathogenic threat to animals, including humans and fish, especially within the host mucosal layers [50–52]. However, the oysters harvested for this study exhibited no obvious signs of clinical pathology. Oyster-associated *Mycoplasma* may instead exhibit a commensal-like lifestyle. In fact, some *Mycoplasma* spp. may represent a potential benefit for their hosts [53, 54].

Bacteria within the family *Spirochaetaceae*, have also been identified in this study and through *in situ* analysis as members of a potential core microbiome family in oysters [15, 47–49, 55–57]. The Spirochaete *Cristispira* genus is commonly associated with mollusks, including the Eastern oyster, where it is present on the crystalline style of oysters used for feeding and may represent a core genus [58–60]. If *Cristispira* is indeed the dominant unidentified *Spirochaetaceae* genus observed in this analysis, then it may represent a commensal of the Eastern oyster.

Although some core bacterial families appear to be capable of maintaining their presence in the oyster microbiome independent of seasonal temperature changes, there are also other organisms that display clear seasonal relative abundance fluctuations. For example, there is an increased relative abundance of Cyanobacteria, when the water column is warmer, and, conversely, an increased relative abundance of Campilobacterota (Fig 2 and S2 Fig) when the water column is colder. The dominant *Cyanobiaceae* organism identified here, *Cyanobium* sp., is capable of producing cyanotoxins and can be associated with cyanobacterial blooms [61–63]. Hazardous algal blooms typically occur during the summer when the water temperature is higher and can present harm to humans and marine life. For example, higher levels of *Cyanobium* sp. within the water column may influence higher incidence of potentially pathogenic *Vibrio* spp. [64].

Bacterial communities present in August oysters had more unique taxa than the other time points (Fig 3) and the overall diversity increased significantly during August (Table 1). Specifically, the number of ASVs that were observed in the August oysters increased in comparison to the other time points; this indicates new taxa have been incorporated. The presence of high levels of Cyanobacteria within the bacterial community may be an important contributing factor driving the diversity increase. Importantly, the unique August taxa did not dominate the bacterial community, as the evenness indicates an even representation by all taxa. This suggests an increase in the total bacterial numbers during August, though this would need to be further quantitated.

The phylum Campilobacterota was primarily represented by *Arcobacteriaceae* in the oysters harvested from colder water. *Pseudoarcobacter* (S2 Table), recently reclassified from the genus *Arcobacter* [65] was a dominant taxon. Though little is published about the *Pseudoarcobacter* genus, the *Arcobacter* genus has been identified in other shellfish including as a common commensal of the oyster microbiota [56, 66, 67]. *Arcobacter* also includes several species known to be pathogenic to humans [68–70]. The relative abundance of *Arcobacteriaceae* in the oyster microbiome was higher in the colder than the warmer months (Fig 2 and S2 Fig), similar to other seasonal patterns of specific *Arcobacteriaceae* spp. [71]. Without knowing the absolute density and species identity of *Arcobacteriaceae* within the oysters across seasons, it is difficult to determine if these taxa might represent a possible pathogenic threat to their hosts and other susceptible animals. However, the data from this study suggest that bacteria within the *Arcobacteriaceae* family are commensal members of the oyster microbiome when the surrounding water column is colder (i.e., November and February).

The year over year analysis (February 2020 compared to February 2021) indicated a decreasing trend in the overall diversity (Table 1), however, the taxa present in these oysters appeared more similar than to other time points (Fig 3). The lower overall diversity may be correlated to the lower temperature in the Chesapeake Bay area during the February 2021 harvest. This phenomenon has been observed in other bivalves showing a decrease in microbial diversity during periods of decreased temperatures [72]. Importantly, the similarities between the two harvests, February 2020 and February 2021, demonstrate an annual pattern in the microbial community composition.

## Conclusion

Overall, the bacterial communities in the microbiome of oysters subjected to similar post-processing conditions were comprised of bacteria that exhibited fluctuating relative abundances related to harvest season temperature, as indicated by their variable relative abundances. However, some organisms, especially the families *Mycoplasmataceae* and *Spirochaetaceae*, remained at a consistently high relative abundance regardless of the harvest season. These organisms are likely members of the core microbiome of consumer-ready Eastern oysters.

## Supporting information

S1 Fig Phylum-level relative abundances within the Eastern oyster bacterial community across time on an individual animal basis. Eastern oysters were collected quarterly over a year (n = 18 oysters per season) including a second harvest one year following the first harvest. Harvest dates included (A) February 2020, (B) June, (C) August, (D) November, and (E) February 2021. The 16S rRNA gene V4 region was sequenced from the oyster homogenates wherein the bacterial communities were examined. The top phyla comprising at least 95% of the total bacterial community for all treatment groups are represented in the plot for each animal.
(TIFF)

S2 Fig Family-level relative abundances within the Eastern oyster bacterial community across time on an individual animal basis. Eastern oysters were collected quarterly over a year (n = 18 oysters per season) including a second harvest one year following the first harvest. Harvest dates included (A) February 2020, (B) June, (C) August, (D) November, and (E) February 2021. The 16S rRNA gene V4 region was sequenced from the oyster homogenates wherein the bacterial communities were examined. The top families comprising at least 95% of the total bacterial community for all treatment groups are represented in the plot for each animal.
(TIFF)

S3 Fig (TIFF)

S1 Table Oyster harvest location buoy data. (DOCX)

S2 Table ASV relative abundance table. (DOCX)
