# Supplementary figures and images for: Analysis of the core bacterial community associated with consumer-ready Eastern oysters (Crassostrea virginica)
Source: PLoS One. 2023 Feb 22;18(2):e0281747. doi: 10.1371/journal.pone.0281747 (PMC9946220; doi:10.1371/journal.pone.0281747)

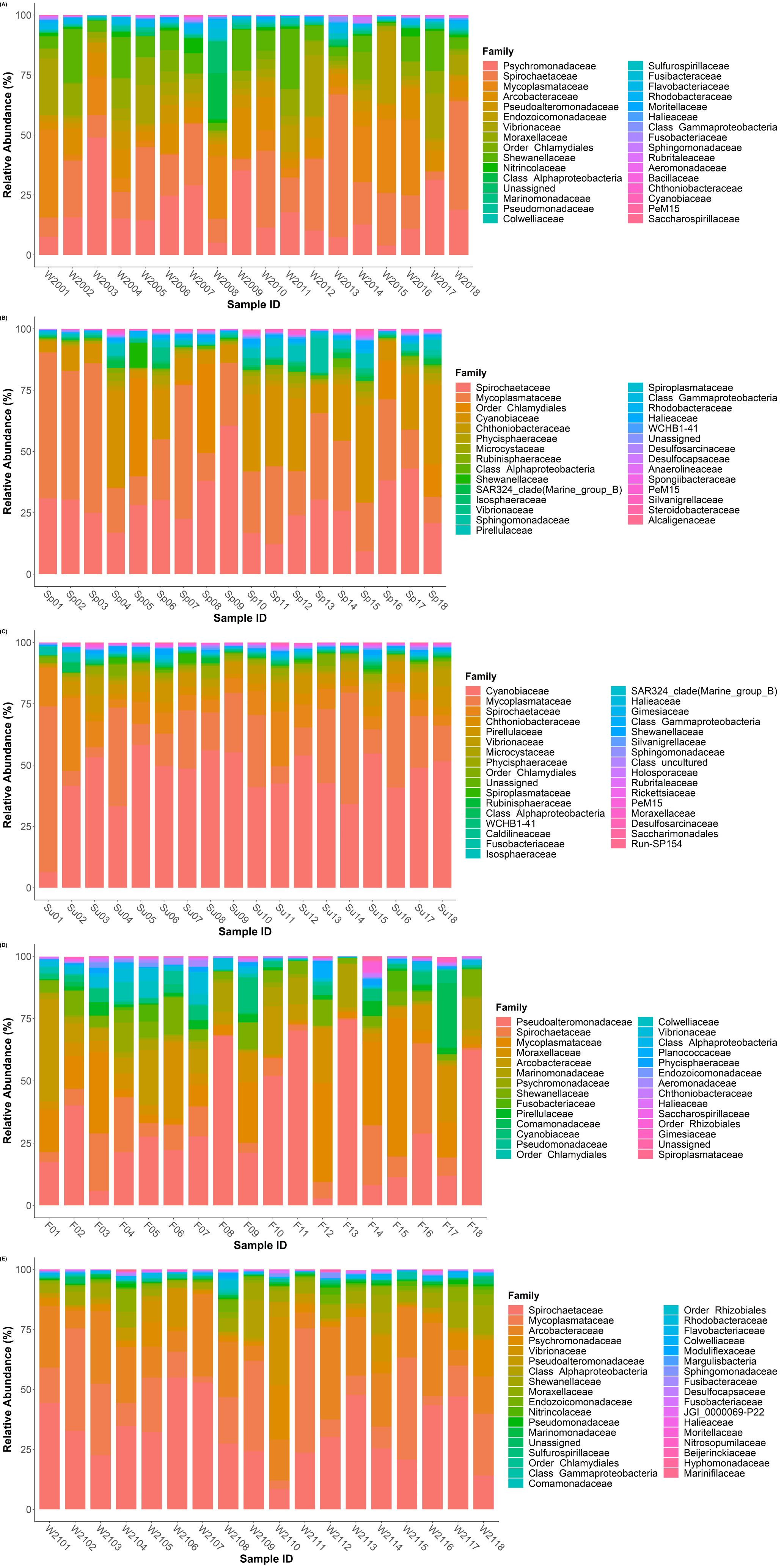

Supplement: S2 Fig — Eastern oysters were collected quarterly over a year (n = 18 oysters per season) including a second harvest one year following the first harvest. Harvest dates included (A) February 2020, (B) June, (C) August, (D) November, and (E) February 2021. The 16S rRNA gene V4 region was sequenced from the oyster homogenates wherein the bacterial communities were examined. The top families comprising at least 95% of the total bacterial community for all treatment groups are represented in the plot for each animal. (TIFF) [file pone.0281747.s002.tiff]

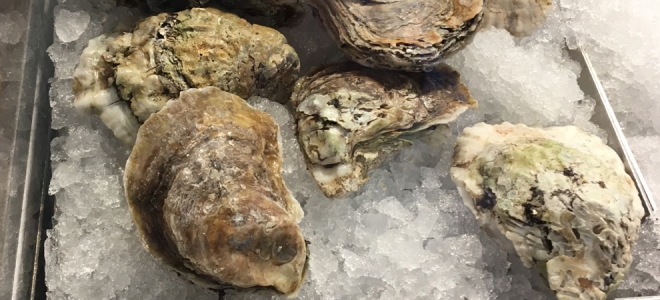

Supplement: S3 Fig — (TIFF) [file pone.0281747.s003.tiff]
