# Supplementary material for: Analysis of the core bacterial community associated with consumer-ready Eastern oysters (Crassostrea virginica)
Source: PLoS One. 2023 Feb 22;18(2):e0281747. doi: 10.1371/journal.pone.0281747 (PMC9946220; doi:10.1371/journal.pone.0281747)
Supplement: S1 Table — (DOCX) [file pone.0281747.s004.docx]

**S1 Table. Oyster harvest location buoy data.**

| Sampling Period | Purchase date | Buoy location name | Buoy location coordinates^1^ | Water Temperature (°C) | Salinity (PSU^2^) | Dissolved Oxygen (mL O_2_/L) |
| --- | --- | --- | --- | --- | --- | --- |
| February (2020) | 2/28/2020 | Potomac (PL) | 38.033, -76.337 | 6.00 | 14.7 | 17.4 |
| June | 6/16/2020 | Gooses Reef (GR) | 38.556, -76.415 | 22.7 | 10.4 | N/A |
| August | 8/26/2020 | Gooses Reef (GR) | 38.556, -76.415 | 26.8 | 13.3 | 10.83 |
| November | 11/23/2020 | Gooses Reef (GR) | 38.556, -76.415 | 16.4 | 16.1 | 9.49 |
| February (2021) | 2/23/2021 | Potomac (PL) | 38.033, -76.337 | 3.33 | 12.3 | 7.73 |

^1^ Information retrieved from NOAA’s Chesapeake Bay Interpretive Buoy System [20]; available data taken from closest buoy to harvest location

^2^ Practical salinity unit (PSU)
