# Supplementary material for: Analysis of the core bacterial community associated with consumer-ready Eastern oysters (Crassostrea virginica)
Source: PLoS One. 2023 Feb 22;18(2):e0281747. doi: 10.1371/journal.pone.0281747 (PMC9946220; doi:10.1371/journal.pone.0281747)
Supplement: S2 Table — (DOCX) [file pone.0281747.s005.docx]

**S2 Table.** ASV relative abundance table.

| **Phylogeny** | | | **Relative abundance (%)^1^** | | | | | |
| --- | --- | --- | --- | --- | --- | --- | --- | --- |
| **Phylum** | **Family** | **Organism name** | **February 2020** | **June** | **August** | **November** | **February 2021** | **Average** |
| Spirochaetota | *Spirochaetaceae* | ***Spirochaetaceae*** | 18.339 ± 0.088 | 24.742 ± 0.102 | 7.979 ± 0.039 | 9.125 ± 0.050 | 30.003 ± 0.121 | 18.038 |
| Firmicutes | *Mycoplasmataceae* | ***Mycoplasma* sp.** | 10.373 ± 0.091 | 23.920 ± 0.153 | 11.116 ± 0.099 | 12.331 ± 0.097 | 19.090 ± 0.128 | 15.366 |
| Cyanobacteria | *Cyanobiaceae* | **Cyanobium_PCC-6307** | 0.089 ± 0.000 | 14.251 ± 0.061 | 39.849 ± 0.147 | 1.708 ± 0.008 | N/A | 11.179 |
| Proteobacteria | *Pseudo-alteromonadaceae* | ***Pseudoalteromonas* sp.** | 3.756 ± 0.031 | N/A | N/A | 30.606 ± 0.308 | 1.795 ± 0.020 | 7.231 |
| Campilobacterota | *Arcobacteraceae* | ***Pseudarcobacter* sp.** | 6.241 ± 0.055 | N/A | N/A | 0.715 ± 0.010 | 17.008 ± 0.127 | 4.793 |
| Proteobacteria | *Psychromonadaceae* | ***Psychromonas* sp.** | 17.058 ± 0.110 | N/A | 0.124 ± 0.001 | 1.259 ± 0.018 | 4.833 ± 0.041 | 4.655 |
| Firmicutes | *Mycoplasmataceae* | ***Mycoplasmataceae*** | N/A | 3.671 ± 0.048 | 10.239 ± 0.155 | 1.168 ± 0.033 | N/A | 3.016 |
| Proteobacteria | *Moraxellaceae* | ***Psychrobacter* sp.** | 5.115 ± 0.052 | N/A | 0.144 ± 0.002 | 5.695 ± 0.091 | 2.101 ± 0.028 | 2.611 |
| Proteobacteria | *Vibrionaceae* | ***Aliivibrio* sp.** | 6.752 ± 0.100 | N/A | N/A | 0.535 ± 0.013 | 3.779 ± 0.096 | 2.213 |
| Campilobacterota | *Arcobacteraceae* | ***Arcobacteraceae*** | 1.981 ± 0.021 | N/A | N/A | 6.261 ± 0.125 | 1.773 ± 0.019 | 2.003 |
| Verrucomicrobiota | Order Chlamydiales | **Order Chlamydiales** | 1.510 ± 0.041 | 5.525 ± 0.151 | 0.775 ± 0.018 | 0.652 ± 0.022 | 0.271 ± 0.006 | 1.747 |
| Proteobacteria | *Shewanellaceae* | ***Shewanella frigidimarina*** | 5.481 ± 0.058 | N/A | N/A | N/A | 2.870 ± 0.021 | 1.670 |
| Proteobacteria | *Endozoico-monadaceae* | ***Endozoicomonas* sp.** | 3.720 ± 0.089 | N/A | N/A | 0.304 ± 0.007 | 0.968 ± 0.016 | 0.998 |
| Proteobacteria | *Shewanellaceae* | ***Shewanella baltica*** | 0.713 ± 0.012 | N/A | 0.302 ± 0.004 | 2.782 ± 0.032 | 1.157 ± 0.007 | 0.991 |
| Spirochaetota | *Spirochaetaceae* | **Spirochaeta_2** | 1.453 ± 0.025 | 1.084 ± 0.018 | 0.547 ± 0.007 | 0.280 ± 0.007 | 1.430 ± 0.030 | 0.959 |
| Verrucomicrobiota | *Chthoniobacteraceae* | **LD29** | 0.082 ± 0.001 | 1.486 ± 0.010 | 2.732 ± 0.014 | 0.279 ± 0.003 | N/A | 0.916 |
| Proteobacteria | *Marinomonadaceae* | ***Marinomonas primoryensis*** | 0.540 ± 0.004 | N/A | N/A | 3.206 ± 0.054 | 0.512 ± 0.004 | 0.852 |
| Planctomycetota | *Pirellulaceae* | **marine metagenome** | N/A | 0.940 ± 0.005 | 1.740 ± 0.007 | 1.503 ± 0.008 | 0.057 ± 0.000 | 0.848 |
| Proteobacteria | Class Alphaproteobacteria | **Class Alpha-proteobacteria** | 1.147 ± 0.028 | 0.602 ± 0.005 | 0.452 ± 0.005 | 0.379 ± 0.004 | 1.426 ± 0.026 | 0.801 |
| Planctomycetota | *Phycisphaeraceae* | **CL500-3** | N/A | 1.414 ± 0.007 | 1.710 ± 0.005 | 0.672 ± 0.004 | N/A | 0.759 |
| Proteobacteria | *Shewanellaceae* | ***Shewanella denitrificans*** | 1.114 ± 0.012 | N/A | N/A | 1.534 ± 0.013 | 0.868 ± 0.007 | 0.703 |
| Proteobacteria | *Vibrionaceae* | ***Vibrio mediterranei*** | N/A | 0.809 ± 0.015 | 1.788 ± 0.023 | 0.708 ± 0.008 | N/A | 0.661 |
| Proteobacteria | *Shewanellaceae* | ***Shewanella aestuarii*** | 1.629 ± 0.014 | N/A | 0.187 ± 0.005 | 0.303 ± 0.004 | 0.663 ± 0.005 | 0.556 |
| Unassigned Phyla | Unassigned | **Unassigned** | 1.050 ± 0.019 | 0.241 ± 0.001 | 0.661 ± 0.003 | 0.178 ± 0.001 | 0.375 ± 0.005 | 0.501 |
| Cyanobacteria | *Cyanobiaceae* | ***Synechococcus* sp.** | N/A | 1.238 ± 0.011 | 1.039 ± 0.005 | N/A | N/A | 0.455 |
| Proteobacteria | *Nitrincolaceae* | ***Neptunomonas* sp.** | 1.174 ± 0.017 | N/A | N/A | N/A | 0.792 ± 0.009 | 0.393 |
| Cyanobacteria | *Microcystaceae* | ***Merismopedia* sp.** | N/A | 1.047 ± 0.011 | 0.876 ± 0.005 | N/A | N/A | 0.385 |
| Cyanobacteria | *Cyanobiaceae* | **Synechococcus_CC9902** | N/A | 0.910 ± 0.008 | 0.247 ± 0.002 | 0.665 ± 0.011 | N/A | 0.364 |
| Proteobacteria | *Pseudomonadaceae* | ***Pseudomonas* sp.** | 0.485 ± 0.004 | N/A | N/A | 0.685 ± 0.014 | 0.600 ± 0.006 | 0.354 |
| Proteobacteria | *Comamonadaceae* | ***Aquabacterium* sp.** | N/A | N/A | N/A | 1.385 ± 0.066 | 0.262 ± 0.004 | 0.329 |
| Planctomycetota | *Pirellulaceae* | ***Pirellula* sp.** | 0.068 ± 0.000 | 0.351 ± 0.001 | 1.179 ± 0.003 | N/A | N/A | 0.320 |
| Planctomycetota | *Pirellulaceae* | ***Pirellulaceae*** | N/A | 0.288 ± 0.002 | 1.057 ± 0.006 | 0.167 ± 0.001 | N/A | 0.302 |
| Proteobacteria | *Moraxellaceae* | ***Acinetobacter* sp.** | N/A | N/A | N/A | 1.430 ± 0.061 | 0.077 ± 0.001 | 0.301 |
| SAR324_clade (Marine_group_B) | SAR324_clade (Marine_group_B) | **SAR324_clade (Marine_group_B)** | N/A | 1.080 ± 0.004 | 0.416 ± 0.001 | N/A | N/A | 0.299 |
| Planctomycetota | *Pirellulaceae* | ***Rhodopirellula* sp.** | N/A | 0.413 ± 0.003 | 1.054 ± 0.005 | N/A | N/A | 0.293 |
| Actinobacteriota | PeM15 | **PeM15** | 0.090 ± 0.000 | 0.542 ± 0.001 | 0.408 ± 0.001 | 0.362 ± 0.001 | N/A | 0.280 |
| Proteobacteria | *Halieaceae* | ***Halioglobus* sp.** | 0.189 ± 0.003 | 0.583 ± 0.007 | 0.277 ± 0.002 | 0.244 ± 0.002 | 0.104 ± 0.001 | 0.279 |
| Cyanobacteria | *Cyanobiaceae* | ***Cyanobium* sp.** | N/A | 0.442 ± 0.004 | 0.322 ± 0.001 | 0.623 ± 0.010 | N/A | 0.277 |
| Proteobacteria | *Vibrionaceae* | ***Vibrio* sp.** | N/A | 0.173 ± 0.004 | 0.169 ± 0.002 | 0.959 ± 0.020 | N/A | 0.260 |
| Fusobacteriota | *Fusobacteriaceae* | ***Psychrilyobacter* sp.** | 0.162 ± 0.003 | N/A | N/A | 0.971 ± 0.030 | 0.165 ± 0.004 | 0.260 |
| Proteobacteria | Class Gamma-proteobacteria | **Class Gamma-proteobacteria** | 0.187 ± 0.001 | 0.364 ± 0.001 | 0.274 ± 0.001 | 0.152 ± 0.000 | 0.282 ± 0.002 | 0.252 |
| Proteobacteria | *Sphingomonadaceae* | ***Sphingoaurantiacus* sp.** | 0.143 ± 0.008 | 0.799 ± 0.041 | 0.212 ± 0.004 | N/A | 0.076 ± 0.004 | 0.246 |
| Firmicutes | *Spiroplasmataceae* | ***Spiroplasma* sp.** | N/A | 0.390 ± 0.009 | 0.611 ± 0.012 | 0.155 ± 0.005 | N/A | 0.231 |
| Bacteroidota | *Flavobacteriaceae* | ***Flavobacterium* sp.** | 0.796 ± 0.007 | N/A | N/A | N/A | 0.293 ± 0.004 | 0.218 |
| Proteobacteria | *Marinomonadaceae* | ***Marinomonas* sp.** | N/A | N/A | N/A | 1.074 ± 0.004 | N/A | 0.215 |
| Proteobacteria | *Rhodobacteraceae* | ***Rhodobacteraceae*** | 0.363 ± 0.004 | 0.389 ± 0.006 | N/A | N/A | 0.191 ± 0.003 | 0.189 |
| Planctomycetota | *Isosphaeraceae* | ***Isosphaeraceae*** | N/A | 0.496 ± 0.004 | 0.335 ± 0.002 | N/A | N/A | 0.166 |
| Proteobacteria | *Shewanellaceae* | ***Shewanella amazonensis*** | N/A | 0.574 ± 0.029 | 0.204 ± 0.006 | N/A | N/A | 0.156 |
| Planctomycetota | *Rubinisphaeraceae* | ***Planctomicrobium* sp.** | N/A | 0.617 ± 0.004 | 0.137 ± 0.000 | N/A | N/A | 0.151 |
| Verrucomicrobiota | WCHB1-41 | **WCHB1-41** | N/A | 0.253 ± 0.002 | 0.499 ± 0.002 | N/A | N/A | 0.150 |
| Proteobacteria | *Aeromonadaceae* | ***Oceanisphaera* sp.** | 0.092 ± 0.001 | N/A | N/A | 0.595 ± 0.004 | 0.056 ± 0.000 | 0.149 |
| Planctomycetota | *Gimesiaceae* | ***Gimesiaceae*** | N/A | N/A | 0.529 ± 0.001 | 0.200 ± 0.002 | N/A | 0.146 |
| Chloroflexi | *Anaerolineaceae* | ***Anaerolineaceae*** | N/A | 0.382 ± 0.001 | 0.302 ± 0.000 | N/A | N/A | 0.137 |
| Proteobacteria | *Shewanellaceae* | ***Shewanella* sp.** | 0.221 ± 0.004 | N/A | N/A | 0.449 ± 0.004 | N/A | 0.134 |
| Desulfobacterota | *Desulfosarcinaceae* | **Sva0081_sediment_group** | N/A | 0.275 ± 0.001 | 0.212 ± 0.001 | 0.163 ± 0.001 | N/A | 0.130 |
| Proteobacteria | *Colwelliaceae* | ***Colwellia* sp.** | 0.461 ± 0.007 | N/A | N/A | N/A | 0.176 ± 0.002 | 0.127 |
| Campilobacterota | *Sulfurospirillaceae* | ***Sulfurospirillum* sp.** | 0.352 ± 0.006 | N/A | N/A | N/A | 0.284 ± 0.004 | 0.127 |
| Proteobacteria | *Colwelliaceae* | ***Colwellia beringensis*** | N/A | N/A | N/A | 0.621 ± 0.022 | N/A | 0.124 |
| Planctomycetota | *Rubinisphaeraceae* | ***Rubinisphaeraceae*** | N/A | N/A | 0.593 ± 0.003 | N/A | N/A | 0.119 |
| Proteobacteria | *Shewanellaceae* | ***Shewanella colwelliana*** | 0.102 ± 0.002 | N/A | N/A | 0.436 ± 0.006 | N/A | 0.108 |
| Bacteroidota | *Flavobacteriaceae* | ***Flavobacterium tegetincola*** | 0.218 ± 0.003 | N/A | N/A | N/A | 0.313 ± 0.003 | 0.106 |
| Proteobacteria | *Shewanellaceae* | ***Shewanella livingstonensis*** | 0.194 ± 0.001 | N/A | N/A | 0.324 ± 0.005 | N/A | 0.104 |
| Firmicutes | *Fusibacteraceae* | ***Fusibacter* sp.** | 0.340 ± 0.006 | N/A | N/A | N/A | 0.143 ± 0.001 | 0.097 |
| Fusobacteriota | *Fusobacteriaceae* | ***Fusobacterium* sp.** | N/A | N/A | 0.349 ± 0.013 | N/A | 0.089 ± 0.003 | 0.088 |
| Proteobacteria | *Vibrionaceae* | ***Photobacterium* sp.** | 0.223 ± 0.002 | N/A | N/A | 0.152 ± 0.004 | 0.058 ± 0.002 | 0.087 |
| Proteobacteria | Order Rhizobiales | **Order Rhizobiales** | N/A | N/A | N/A | 0.226 ± 0.012 | 0.206 ± 0.010 | 0.086 |
| Proteobacteria | *Moritellaceae* | ***Moritella* sp.** | 0.271 ± 0.002 | N/A | N/A | N/A | 0.141 ± 0.001 | 0.082 |
| Proteobacteria | *Rhodobacteraceae* | ***Marivita* sp.** | 0.182 ± 0.002 | 0.228 ± 0.002 | N/A | N/A | N/A | 0.082 |
| Desulfobacterota | *Desulfocapsaceae* | ***Desulfocapsaceae*** | 0.069 ± 0.001 | 0.185 ± 0.001 | N/A | N/A | 0.142 ± 0.001 | 0.079 |
| Chloroflexi | *Caldilineaceae* | ***Caldilineaceae*** | N/A | N/A | 0.375 ± 0.002 | N/A | N/A | 0.075 |
| Firmicutes | *Planococcaceae* | ***Planococcaceae*** | N/A | N/A | N/A | 0.365 ± 0.015 | N/A | 0.073 |
| Proteobacteria | *Sphingomonadaceae* | ***Sphingomonas* sp.** | N/A | 0.159 ± 0.006 | N/A | N/A | 0.203 ± 0.007 | 0.072 |
| Bdellovibrionota | *Silvanigrellaceae* | ***Silvanigrellaceae*** | N/A | 0.147 ± 0.002 | 0.213 ± 0.002 | N/A | N/A | 0.072 |
| Bacteroidota | *Flavobacteriaceae* | ***Flavobacterium jumunjinense*** | 0.297 ± 0.007 | N/A | N/A | N/A | 0.060 ± 0.001 | 0.071 |
| Planctomycetota | *Pirellulaceae* | ***Blastopirellula* sp.** | N/A | 0.348 ± 0.002 | N/A | N/A | N/A | 0.070 |
| Verrucomicrobiota | DEV007 | **DEV007** | N/A | 0.192 ± 0.000 | 0.132 ± 0.000 | N/A | N/A | 0.065 |
| Proteobacteria | *Saccharospirillaceae* | ***Oleispira* sp.** | 0.069 ± 0.001 | N/A | N/A | 0.247 ± 0.003 | N/A | 0.063 |
| Verrucomicrobiota | *Rubritaleaceae* | ***Luteolibacter* sp.** | 0.125 ± 0.001 | N/A | 0.187 ± 0.001 | N/A | N/A | 0.062 |
| Planctomycetota | *Pirellulaceae* | **bacterium enrichment** | N/A | N/A | 0.308 ± 0.000 | N/A | N/A | 0.062 |
| Proteobacteria | B2M28 | **B2M28** | N/A | 0.132 ± 0.001 | 0.138 ± 0.001 | N/A | N/A | 0.054 |
| Proteobacteria | Run-SP154 | **Run-SP154** | N/A | 0.123 ± 0.001 | 0.125 ± 0.001 | N/A | N/A | 0.050 |
| Proteobacteria | *Holosporaceae* | ***Holosporaceae*** | N/A | N/A | 0.203 ± 0.001 | N/A | N/A | 0.041 |
| Desulfobacterota | uncultured bacterium | **uncultured bacterium** | N/A | N/A | 0.202 ± 0.001 | N/A | N/A | 0.040 |
| Cyanobacteria | *Microcystaceae* | **Synechocystis_PCC-6803 sp.** | N/A | 0.199 ± 0.002 | N/A | N/A | N/A | 0.040 |
| Bacteroidota | *Flavobacteriaceae* | ***Flavobacteriaceae*** | 0.109 ± 0.001 | N/A | N/A | N/A | 0.084 ± 0.001 | 0.039 |
| Proteobacteria | *Rickettsiaceae* | **Candidatus_Megaira sp.** | N/A | N/A | 0.178 ± 0.001 | N/A | N/A | 0.036 |
| Proteobacteria | *Comamonadaceae* | ***Acidovorax* sp.** | N/A | N/A | N/A | 0.173 ± 0.008 | N/A | 0.035 |
| Margulisbacteria | *Margulisbacteria* | ***Margulisbacteria* sp.** | N/A | N/A | N/A | N/A | 0.170 ± 0.002 | 0.034 |
| Modulibacteria | *Moduliflexaceae* | ***Moduliflexaceae* sp.** | N/A | N/A | N/A | N/A | 0.166 ± 0.005 | 0.033 |
| Proteobacteria | *Halieaceae* | ***Luminiphilus* sp.** | N/A | 0.163 ± 0.002 | N/A | N/A | N/A | 0.033 |
| Proteobacteria | *Spongiibacteraceae* | **BD1-7_clade** | N/A | 0.160 ± 0.001 | N/A | N/A | N/A | 0.032 |
| Proteobacteria | *Chromatiaceae* | ***Chromatiaceae*** | N/A | 0.156 ± 0.001 | N/A | N/A | N/A | 0.031 |
| Campilobacterota | *Arcobacteraceae* | ***Arcobacter nitrofigilis*** | N/A | N/A | N/A | N/A | 0.153 ± 0.004 | 0.031 |
| Proteobacteria | *Steroidobacteraceae* | ***Steroidobacteraceae*** | N/A | 0.136 ± 0.001 | N/A | N/A | N/A | 0.027 |
| Proteobacteria | *Aeromonadaceae* | ***Aeromonas* sp.** | N/A | N/A | N/A | 0.136 ± 0.005 | N/A | 0.027 |
| Patescibacteria | *Saccharimonadales* | ***Saccharimonadales* sp.** | N/A | N/A | 0.135 ± 0.001 | N/A | N/A | 0.027 |
| Proteobacteria | *Halieaceae* | ***Halieaceae*** | N/A | 0.131 ± 0.001 | N/A | N/A | N/A | 0.026 |
| Actinobacteriota | *Mycobacteriaceae* | ***Mycobacterium* sp.** | N/A | N/A | N/A | 0.130 ± 0.002 | N/A | 0.026 |
| Proteobacteria | *Alcaligenaceae* | ***Bordetella* sp.** | N/A | 0.129 ± 0.001 | N/A | N/A | N/A | 0.026 |
| Proteobacteria | *Shewanellaceae* | ***Shewanella loihica*** | N/A | 0.126 ± 0.003 | N/A | N/A | N/A | 0.025 |
| Desulfobacterota | *Desulfocapsaceae* | ***Desulforhopalus* sp.** | N/A | 0.124 ± 0.001 | N/A | N/A | N/A | 0.025 |
| Bacteroidota | *Flavobacteriaceae* | ***Lutibacter* sp.** | 0.120 ± 0.001 | N/A | N/A | N/A | N/A | 0.024 |
| Proteobacteria | *Moraxellaceae* | ***Psychrobacter maritimus*** | 0.115 ± 0.001 | N/A | N/A | N/A | N/A | 0.023 |
| Patescibacteria | JGI_0000069-P22 | **JGI_0000069-P22** | N/A | N/A | N/A | N/A | 0.112 ± 0.001 | 0.022 |
| Crenarchaeota | *Nitrosopumilaceae* | **Candidatus_Nitrosopumilus sp.** | N/A | N/A | N/A | N/A | 0.093 ± 0.001 | 0.019 |
| Firmicutes | *Bacillaceae* | ***Bacillus* sp.** | 0.087 ± 0.002 | N/A | N/A | N/A | N/A | 0.017 |
| Proteobacteria | *Beijerinckiaceae* | ***Methylobacterium-Methylorubrum* sp.** | N/A | N/A | N/A | N/A | 0.079 ± 0.003 | 0.016 |
| Proteobacteria | *Hyphomonadaceae* | **Alpha-proteobacterium** | N/A | N/A | N/A | N/A | 0.070 ± 0.000 | 0.014 |
| Verrucomicrobiota | *Rubritaleaceae* | ***Persicirhabdus* sp.** | 0.069 ± 0.001 | N/A | N/A | N/A | N/A | 0.014 |
| Bacteroidota | *Marinifilaceae* | ***Marinifilaceae*** | N/A | N/A | N/A | N/A | 0.064 ± 0.001 | 0.013 |

^1^ Error is represented by the mean relative abundance ± standard error of the mean (SEM) of 18 oysters
